# Fracture fixation in polytraumatized patients—From an interdisciplinary early total/appropriate care to the safe definitive surgery concept

**DOI:** 10.3389/fmed.2024.1362986

**Published:** 2024-04-19

**Authors:** Yannik Kalbas, Sandro-Michael Heining, Anne Kaiser, Felix Karl-Ludwig Klingebiel, Roman Pfeifer, Guido A. Wanner, Hans-Christoph Pape

**Affiliations:** ^1^Department of Trauma Surgery, University Hospital Zurich, University of Zurich, Zurich, Switzerland; ^2^Department of Anesthesia, University Hospital Zurich, University of Zurich, Zurich, Switzerland; ^3^Department of Spine and Trauma Surgery, Bethanien Hospital, Zurich, Switzerland

**Keywords:** polytrauma, safe definitive surgery, fracture management, borderline patient, multiply injured patients

## Abstract

The strategies for the timing of fracture fixation in polytrauma patients have changed with improvements in resuscitation and patient assessment. Specifically, the criteria for damage control have been formulated, and more precise parameters have been found to determine those patients who can safely undergo primary definitive fixation of major fractures. Our current recommendations are supported by objective and data-based criteria and development groups. Those were validated and compared to existing scores. This review article introduces the concept of “safe definitive surgery” and provides an update on the parameters used to clear patients for timely fixation of major fractures.

## Introduction

After most surgeons avoided performing major surgeries on patients with questionable clinical status, our group determined the clinical parameters that are relevant for the prediction of complications. This analysis led to the application of early definitive fracture fixation, starting within 24 h after injury. As this practice implies the exclusion of risk factors, it was named “safe definitive surgery” (SDS).

Following a development phase, the criteria adopted in an independent database proved that, after sorting out the exceptional cases requiring damage control, it is of value to allow for fixing fractures in a timely fashion, most of them within 24 h after admission. One of the milestones indicating the change in management of treatment was obvious in a recent survey among international surgeons. The survey indicated that a fixed timeline is no longer followed. Instead, the fixation strategy follows the stability of parameters, and fixation within the 24-h limit continues to prevail.

The SDS was developed based on parameters that currently appear to be required to allow for a timely fixation of major fractures, with respect to the patient's physiological response. The criteria have been summarized in a review article in 2005 (1), which has recently been updated (2). Our article summarizes the key strategies applied, such as (a) the inclusion of set resuscitation criteria (3); (b) the application importance of utilizing multiple physiological parameters for the assessment of patients; (c) the value of serial reassessments over the course of resuscitation (4–7); and (d) the timely fixation of patients within the 24-h timeline (8).

## Methods

### Development group

In this review, the development of several treatment strategies was summarized and supported by data from a large database (2). The data were stratified into three different time periods. In group 1, 867 patients (23.6%) were treated prior to 2001 (before the introduction of damage control techniques). Groups 2 and 3 consisted of a total of 2,801 patients, of which 1,262 patients (45.1%) were treated between 2003 and 2008 [the incorporation of damage control techniques for major fractures (Group 2)] and 1,539 patients (54.9%) were treated after 2009 [after introducing changes in nonsurgical management, e.g., after the introduction of transfusion and fluid management guidelines (Group 3)]. These three groups constituted the development group.

### Validation group

The validation group consisted of another database, which was used to compare several existing assessment scores. The database utilized the parameters of 3,888 patients who were treated before 2022. It compared four different scales: (a) the so-called clinical grading system (CGS, based on a simple review of parameters in 2005); (b) the modified clinical grading system (mCGS, a modification of the first review based on 750 patients collected from a database in Cleveland, which featured fewer parameters than the CGS); (c) the early appropriate care (EAC) protocol (based on 1,443 patients who were treated in Cleveland between 1999 and 2006); and (d) the polytrauma grading score (PTGS) (11,436 patients who were treated before 2020 from the German Trauma Registry).

### Outcome parameters

In the development group, the parameters considered were mortality rate, ventilation time, intensive care unit (ICU) stay and complications, such as the incidence of pneumonia, the incidence of sepsis, death from shock, and death from head trauma.

## Discussion

The time frame to determine fracture care as “early,” “appropriate,” or “delayed” has been under discussion for a long time. In order to allow for safe definitive fixation of major fractures, resuscitation has to be obviously completed. Some authors have argued that completion of resuscitation has been achieved within 24 h after the injury, though this is subject to debate until robust evidence emerges, which includes the absence of vasopressors, the reduction of acid–base status, the normalization of platelet counts and rotational thromboelastometry (ROTEM) values, and the absence of a positive fluid balance (Table 1).

**Table 1:** Criteria for laboratory values and parameters that should normalize within 24 h after injury (e.g., borderline patient with responsive physiology) to allow for safe definitive surgery.

**Parameter group**	**Criteria for normal**	**Duration until normal**
Shock	Vasopress. infusion < 3 ml/h or absent	4–6 h
Acid base changes	2–2.5 mmol/L	12–24 h
Platelet count (ROTEM)	>90.000 or rising (acc. to system)	8–12 h
Fluid balance	I/o ratio balanced without vasopressors	24 h
Severe chest injury	Absence or reduction of lung contusion	24 h

The criteria to distinguish between different time periods of fracture care might be based on several criteria, such as (a) the time elapsed after an injury, (b) the completion of normalization of parameters summarized under “resuscitation,” or (c) convenience aspects (e.g., the availability of operating rooms). The last mentioned criterion has been added recently, and it became evident that having room to take care of acute emergencies is a feasible option to reduce delays caused by technical operating room availability. Regarding the time elapsed after an injury, this criterion has been the major determinant in the transition era when the term “early total care” was coined. Furthermore, it occurred when early fracture care was proven to be more beneficial than waiting a week to 10 days in the fear of the patient developing a systemic fat embolism syndrome. Despite the lack of clear-cut guidelines for resuscitation, mass transfusions or endpoints were considered as clearance for going to the operating room. The authors who advocated that all fractures (major long bones and others) should be stabilized within a time frame did so in the absence of data to support this idea. The development of the criteria above, namely resuscitation guidelines, led to a different method of care, and the variation between 36 and 48 h was evident, even within the same clinical group, as depicted in Table 2.

**Table 2:** The discussion about optimal early definitive care in patients cleared for surgery: Is timing (24/36/48 h) crucial or lab criteria only?

**Author**	**Year**	**Origin country/city**	**Timeline 24 h**	**Concept**	**Lab criteria**
O‘Toole	2009	Baltimore/USA	24	ETC	Resuscitation/lactate (< 2 mmol)
Schreiber	2011	Pittsburgh/leeds	24	DCO	4 categories
Vallier	2013	Cleveland/USA	24	ETC	Lactate (< 4 mmol/L)
Dienstknecht	2013	Aachen/GER	24	DCO	4 categories
Nahm	2014	Cleveland/USA	24	EAC	Lactate (< 4 mmol/L)
Weinberg	2015	Cleveland/USA	36	EAC	–
Pape	2015	Aachen/Ger	< 24	SDS	4 cat
Giannoudis	2016	Leeds/GB	< 24	PRISM	Mult. categories
Vallier	2016	Cleveland/USA	36	EAC	Lactate (< 4 mmol/L)
Childs	2017	Cleveland/USA	24	EAC	Lactate (< 4 mmol/L)
Blockhuis	2017	Utrecht/NL	< 24	SDS	–
Pape	2019	Zurich/SUI	< 24	SDS	4 categories (see Table 5), surgery asap
Volpin	2021	Haifa/ISR	24	DCO/SDS	–
Halvachizadeh	2021	Zurich/SUI	< 24	SDS	4 categories (see Table 5), Surgery asap
Scherer	2022	Zurich/SUI	< 24	SDS	4 categories (see Table 5), surgery asap
Blaesius	2022	Aachen/GER	24	ETC/SDS	PTGS score
Pfeifer	2023	Zurich/SUI	< 24	SDS	4 categories (see Table 5), Surgery asap
Halvachizadeh	In press	Zurich/SUI	< 24	SDS	4 categories (see Table 5), Surgery asap

Variability of lactate threshold levels and recommendations of surgical timing in the orthopedic literature since 2010.

Subsequently, clinical parameters were advocated to control for the effects of resuscitation (3). Among these parameters were acid–base changes and lactate clearance, and the first publication to use the term “lactate clearance” examined lactate levels at 8, 16, 24, 36, and 48 h after injury (2 mmol/L served as the threshold level). The authors clearly concluded that, usually, the survival rate of polytrauma patients with severe hemorrhage was 75%. In these patients, the lactate levels had to be normalized within 24 h (4), that is, a lactate level of 2.0 mmol/L should be achieved, which is in accordance with the majority of the relevant literature (5).

One of the hallmark study series has been popularized by Dezman et al. The authors reported on patients treated between 2010 and 2012 who had the lactate level of >3 mmol/L at admission. The timeline for blood sampling was 24 h post admission. The authors describe a subgroup of patients that normalized their lactate levels within 24 h, and this group was named the “*high clearance”* group. Another group that did not improve their lactate levels within this time frame was named the “*poor clearance”* group. Thus, the timeline of completion of resuscitation is usually 24 h. Along these lines, the authors concluded the superiority of 24 h lactate clearance over using the lactate value only at admission (6, 7).

Kucukdurmaz et al. in their discussion regarding the EAC vs. Damage Control Orthopedics (DCO) approaches examined the 24 h lactate value, which should not exceed more than 2.5 mmol/L, and focused on late respiratory complications (9). Stahel et al. observed that the closure of the abdomen can be performed in close proximity to fracture fixation of the femur, i.e., in one surgical session (10). As mentioned earlier, there have been changes even in the group that developed the EAC protocol, as they initially started at a threshold level of 4 mmol/L of lactate on admission and reduced it to 2.5 mmol/L.

More recently, coagulopathy has been similarly in focus and has become a major determinant of discussing whether a patient is stable, borderline, or unstable (11). Our group has developed reviews to address the issue of threshold levels to separate these clinical entities. Similar trends were followed by Regnier in 2012, when focusing on the value of lactate (5), and by Shapiro et al., who examined 576 trauma patients where the endpoint was mortality (12).

### Timing of fracture fixation in the context of physiological stability

Historically, there appeared to be a consensus on the implementation of an early definitive care approach, and most centers attempted to follow the rules of Bone and Johnson (13).

Later, operating room (OR) availability has been on focus, and many countries have taken the initiatives to develop a “dedicated trauma room” in order to allow for rapid access. Nevertheless, some centers have been cautious and claimed that the completion of resuscitation has to be achieved before fixation. Moreover, surgeon preference was discussed rather than patient criteria after the completion of resuscitation. Therefore, some authors accepted a delay in the fixation of the first major fracture, and timelines changed from 24 to 36 h under many circumstances (Table 2). There has been a development in utilizing different endpoints of resuscitation within departments in the last few years, including acid–base changes along with coagulation and physiological parameters, such as blood pressure, which has led to a considerable improvement of the classification options, as summarized recently (11).

One of the hallmarks of the development against a set timeline has been a survey conducted among experienced surgeons. The surgeons responded by stating that the timing of surgery no longer uses a fixed timeline, as initiated before, but a physiology-based approach is utilized (11). This use of approach is in line with our own concept, as proposed in the SDS protocol, and Prompt-Individualised-Safe Management (PRISM) concept by Giannoudis et al. (14, 15).

### The influence of trauma systems on the team approach and timing of fracture care

The organization of trauma care differs between the US and many regions in Europe (Table 3). These differences concern the rescue crew, where, in the US, trained paramedics usually perform a “scoop and run” approach to bring the patient to the closest hospital. More recently, this approach has been reinforced by the fact that hospital chains have become stronger and taken over the rescue issue by choosing which particular hospital should be served first. These economic principles would overcome the stipulations in the certification processes governed by state regulations. The issues about quality control have not been formally addressed, as the National Trauma Data in the US database does not cover secondary complications.

**Table 3:** Comparison of the U.S. vs. EU trauma system.

**Parameter/time after accident**	**USA**	**EU**
**Rescue**		
Type of training	Trained paramedics 12 M., EMTs Param. exam.	Trained physicians completed residency, then 1 year resc. course, proof of ATLS, or polytrauma course
Max. tx. on scene	Intubation, CPR	Intubation, CPR, chest drain, central line, ultrasound (some)
**Admission**		
ER treatment	Emerg. Med. or Gen surgeon	Shock room leaders Unfallchirurg and anesthesia
Organization of surgical care	Gen. surgeon consults Orthop. trauma	Unfallchirurg
Aftertreatment	Orthopedic trauma surgeon	Unfallchirurg

In Europe, a different approach is adopted. First, a rescue personnel should have completed residency, followed by certain emergency medicine courses and at least one other course, such as Advance Trauma Life Support (ATLS), the Polytrauma course, or the European Trauma Course (16). In Switzerland, these courses are also included in the newly developed trauma surgery education, which requires certain exposure after completion of the surgical or orthopedic residency (https://sgact.ch/schwerpunkt-spez-traumatologie).

The in-hospital treatment is also substantially different. In the US, the multiply injured patient is admitted by a general surgeon, who then consults the orthopedic service in case of fractures (Table 3). In contrast, the admission team in most European health centers consists of both anesthesia and trauma surgery specialists who perform diagnostic procedures in parallel and usually perform an emergency computed tomographic (CT) scan within the first minutes after admission. The certification process to be accepted as a major trauma center includes certain diagnostic criteria, such as the “time to CT scan.” It is one of the important quality criteria, which are reported during the annual feedback conducted at the annual regional trauma congress (https://www.traumaregister-dgu.de/index.php).

These and other factors may be involved in the fact that the German Trauma Registry (TR-DGU) incentivises all level I trauma centers to have the diagnostics completed within 2–3 h and life-threatening procedures performed within the same time frame, including the definitive procedure.

### Current status of decision-making for patients with major fractures according to the SDS concept

The concept of “safe definitive surgery” (SDS) relies on serial measurements of several representative physiological parameters and on the dynamic reevaluation of the patient's physiology during the course of resuscitation and operative interventions (8).

The initial assessment and first treatment measures in the polytrauma patient are highly standardized and follow the principles of ATLS (16). ATLS is a program aimed at restoring the derailment of the patient's physiology, typically caused by either insufficient oxygenation, insufficient perfusion of the end organ, or a combination of both. Although these initial measures have been taken, the timing and sequence of operative procedures are not specified (17). There is a general consensus that definitive operative procedures should be performed once the patient's physiology has been sufficiently restored. However, there is still little agreement on how to reliably quantify the restoration of patient's physiology (18).

The approach used in the past was the ubiquitous application of damage control strategies on the first day and the conversion to a definitive stabilization in the so-called window of opportunity after several days. If applied in an unreflected fashion, however, damage control strategies might lead to relevant restrictions of patient positioning, prolonged immobilization, and delayed definitive surgeries, resulting in an unjustifiable lengthening of hospital stay (19). The concept of early appropriate care (EAC) has been proposed to clear patients for rapid fracture fixation (20). However, this approach included only one aspect of the pathophysiology (acid–base changes) and is dependent on only one measurement (on admission). Several studies have argued that repeat measurements and the inclusion of multiple parameters yield a superior predictive power of unfavorable outcomes. Dezman et al. showed the superior predictive capability of 24 h mortality by utilizing serial lactate measurements and coined the term lactate clearance (6). Moreover, Halvachizadeh et al. have determined that the combination of several parameters, including hemorrhage, coagulation, acid–base status, and soft tissue damage, provide superior predictive power of complications than using only one physiological parameter (21). Indeed, applying the parameters from the Polytrauma Grading Score, which include systolic blood pressure, international normalized ratio (INR), thrombocyte count, base deficit, packed red blood cells, and the new injury severity score (NISS), significantly increased the predictive capabilities for the development of sepsis, pneumonia, and other late complications (21, 22). An overview of several published scores is provided in Table 4.

**Table 4:** Development of scores to determine the state of multiply injured patients on admission, separated by evidence level.

	**Names**	**Level of evidence**	**Pathophysiological changes included**			
			**Shock (Acid/Base)**	**Coagulopathy**	**Hypothermia**	**Soft tissue injury**
Pape, 2005	CGS	Level IV	Multiple	Multiple	Temp.	Multiple
Dienstknecht, 2013	No name	Level III	BD	PTT	–	Multiple
Nahm 2013	mCGS	Level III	Acidosis	Platelets	Temp.	AIS
Vallier, 2013	EAC	Level II	Acidosis	–	–	–
Hildebrand, 2014	PTGS	Level III	BD, pRBC	INR	–	ISS
Halvachizadeh, 2020	SDS concept	Level II	BD, pRBC, BP	PTT, platelets	Temp.	AIS/ISS

BD, base deficit; BP, blood pressure; INR, international normalized ratio; pRBC, packed red blood cells; PTT, partial thromboplastin time.

In view of these considerations, SDS proposes to evaluate patients using a combination of parameters and to perform repeat measurements, allowing a dynamic reassessment based on the response to resuscitation and operative interventions.

The choice of parameters used in SDS is based on the understanding of the pathophysiological posttraumatic response, especially of the interplay of hypothermia, coagulopathy, hemorrhage, and tissue injury (23). An overview of the most relevant parameters is presented in Table 3. These parameters have been shown to adequately estimate the physiological response to severe trauma and resuscitative efforts. They significantly influence the patients' clinical course and have been recently validated by a systematic review, which aimed to identify thresholds that are indicative of a higher rate of adverse outcomes in polytrauma (11).

**Figure 1:**
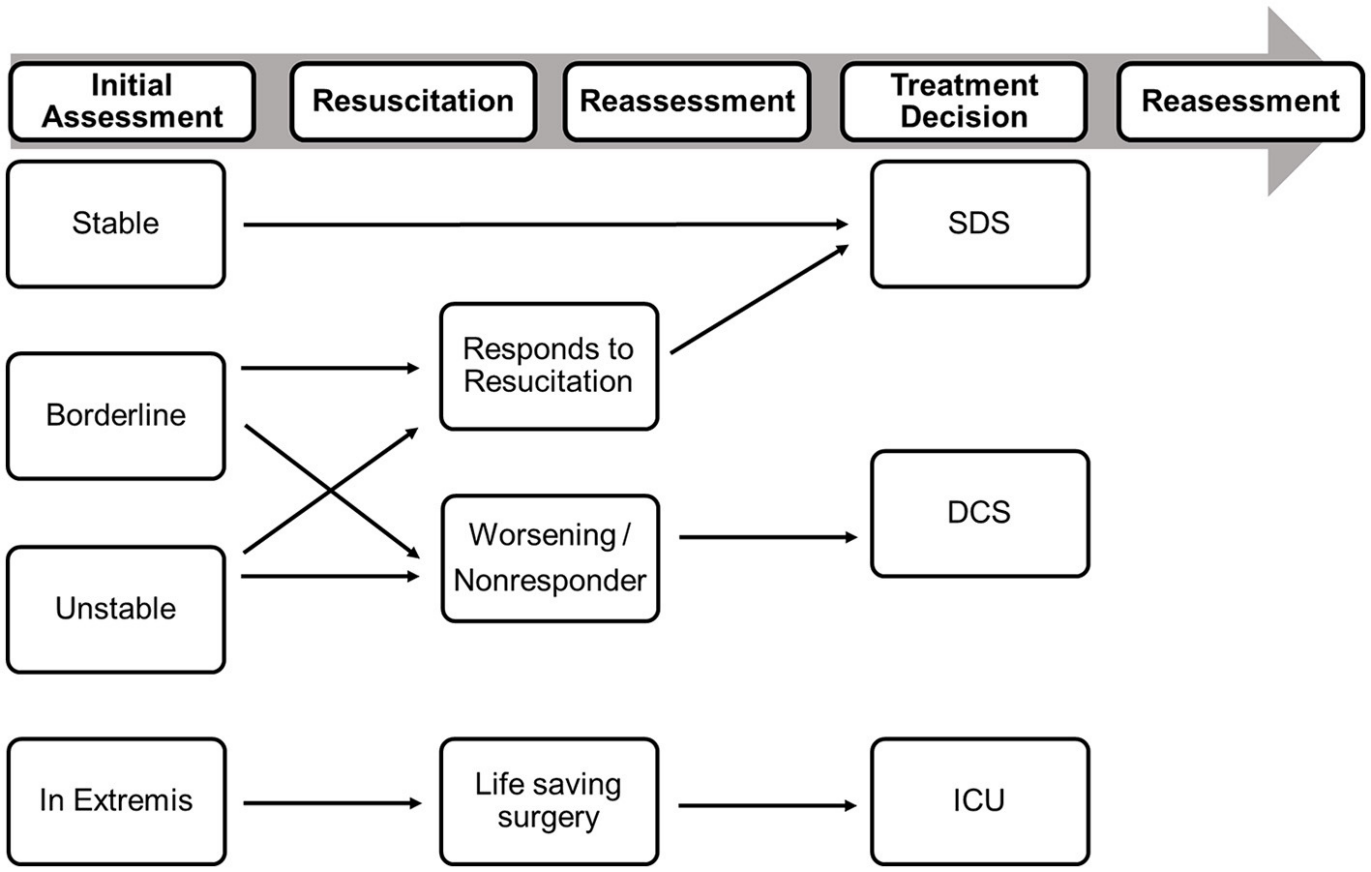
Decision-making in Polytrauma patients should be based on the initial assessment of the patient physiology and on the response to resuscitative measures. DCS, damage control surgery; ICU, intensive care unit [modified from Pape et al. (8)].

Hemorrhage may be identified by systolic blood pressure, lactate, and hemoglobin levels; coagulopathy may be identified by INR or viscoelastic tests ROTEM and hypothermia may be identified by body temperature. There is a recent consensus among leading surgeons (unpublished to date) that the classification of tissue injuries remains challenging and varies between body regions. Traumatic brain injury (TBI) can be evaluated using the intracranial perfusion pressure (ICP), cerebral perfusion pressure (CPP), and the presence of a midline shift, while thoracic tissue trauma can be assessed using the Thorax Trauma Severity Score (TTSS) (24). Abdominal injuries are most frequently graded using the Moore or the American Association for the Surgery of Trauma (AAST) classifications (25). Further parameters that should be considered are the overall injury severity (i.e., NISS), the injury pattern, the number of fractured long bones, and the number of required blood transfusions (26). Based on these parameters, “unstable” or “borderline” stable patients can be identified, and the timing of fracture fixation can be adjusted accordingly (Table 5). It is important to note, however, that these categories are dynamic and that patients can improve or deteriorate depending on their response to resuscitative measures and operative interventions. This process is visualized in Figure 1.

**Table 5:** Threshold levels of parameters to separate stable from borderline patients apply four different categories (20).

**Category**	**Parameter**	**Threshold—borderline**	**Threshold—unstable**
Hemorrhage	SBP (mmHg)	< 100	< 90
	Lactate (mmol/L)	>2	>4
	Hemoglobin (g/dl)	< 9	< 7
	PBRC (on first day)	>2	>8
Hypothermia	Body temperature (°C)	< 35	< 33
Coagulopathy	INR	>1.2	>1.5
ROTEM	Extem CT (s)	>60	>80
	Extem MCF (mm)	< 60	< 45
	Fibtem MCF (mm)	< 12	< 5
TEG	ACT (s)	>110	>128
	MA (mm)	< 60	< 55
	LY30 (%)	>3	>5
**Tissue injury**			
Brain	ICP (mmHg)	>15	>20
	CPP (mmHg)	< 70	< 60
	Midline shift (mm)	>5	≥5
Chest	TTSS	>6	>7
Abdomen	Moore classification	>2	>3

In a study that included 3,668 polytraumatized patients, a significant decrease in early mortality, overall mortality, and complication rates since the introduction of optimized transfusion and fluid management guidelines was observed (2). This report is in line with the survey indicated above, where there is no longer a set timeline, but the stability of parameters is regarded as the endpoint.

In line with these reports, the surgeon panel agreed on the following hierarchy of surgical interventions builds: (a) life-saving operations (i.e., patent airway, pneumothoraces, and uncontrollable hemorrhage); (b) central nervous system (CNS)-saving operations (i.e., severe traumatic brain injury, and spinal cord injury); (c) limb-saving operation (i.e., vascular injuries, mangled extremities, and compartment syndrome); and (d) operations preserving local function and preventing local complications (e.g., open fractures and severe dislocation).

Further considerations should include expected blood loss, post-interventional systemic inflammatory response, potential (pulmonary) complications (e.g., avoid reamed intramedullary nailing in patients with severe chest trauma), patient positioning, duration of immobilization, and pain control. Moreover, the combined operation time should generally not exceed 6 h, and the complexity of fractures needs to be assessed in accordance with the individual surgeons' skills. Finally, it also remains pivotal to evaluate local factors that might prohibit definitive fixation and drive musculoskeletal temporary surgery such as contamination and severe soft tissue trauma (27).

In view of these considerations, the polytrauma section of the European Society for Trauma and Emergency Surgery (ESTES) has led an initiative to introduce a definition for “major fracture(s)” in the multiply injured patient (28, 29). A recent systematic review showed that, over the last decades, the timing of fixation of pelvic and spinal fractures gained importance in the treatment of polytrauma patients, which is likely due to improved diagnostic tools and less invasive operative techniques. Another important finding was that hemodynamic stability And injury-specific factors (e.g., associated soft tissue injuries) have increased in importance over time, while chest injury and TBI have always been important factors in perioperative decision-making (28).

Another recent study presented the results of an international expert opinion questionnaire that focused on factors to be considered to adjust the physiological insult through surgery, coining the term “surgical load.” This study confirmed that surgical sequencing should be performed according to the risk of bleeding, fracture complexity, and the anatomic region. Open surgical procedures as well as surgeries on the trunk, greater articulations, and long bones seem to lead to a higher surgical load than their minimally invasive counterparts or operations on the distal extremities (30).

Nevertheless, there has not yet been a comprehensive grading of the surgical priorities based on the anatomical region of injury. It rather seems that further injury- and patient-specific factors should play a superior role in determining the sequence of operative fixation. This is further emphasized by the recent revision of the abbreviated injury scale, which gives higher scores for fractures if they are open, or associated with severe soft tissue injury (31).

## Conclusion

The understanding of the pathophysiology of patients with polytrauma continues to improve. Besides the physiological effect of the initial traumatic load, this understanding also includes the impact of resuscitative efforts and surgical interventions. The concept of “safe definitive surgery” builds on this knowledge to enable timely and safe fracture fixation in severely injured patients, to be completed within 24 h after admission for patients who do not require a damage control approach. It is important to perform reassessment of patients intraoperatively.

International and multidisciplinary groups of experts are currently preparing consensus statements for fracture fixation in patients with severe concomitant injuries. Another promising approach might be to investigate advanced analytical tools (e.g., proteomic, metabolomic, and lipidomic analyses, and real-time immunofluorescence) in polytrauma patients to further extend the insight into the systemic posttraumatic response and to identify potential new markers for point-of-care resuscitation.

## Author contributions

YK: Data curation, Writing—original draft, Writing—review & editing, Visualization. S-MH: Writing—review & editing, Investigation, Project administration, Validation. AK: Writing—review & editing, Formal analysis, Supervision. FK: Data curation, Visualization, Writing—original draft, Writing—review & editing. RP: Project administration, Writing—review & editing, Visualization. GW: Conceptualization, Methodology, Writing—review & editing, H-CP: Conceptualization, Data curation, Investigation, Writing—original draft, Writing—review & editing.
